# Biochar from Fique Bagasse for Remotion of Caffeine and Diclofenac from Aqueous Solution

**DOI:** 10.3390/molecules25081849

**Published:** 2020-04-17

**Authors:** Yaned Milena Correa-Navarro, Liliana Giraldo, Juan Carlos Moreno-Piraján

**Affiliations:** 1 Departamento de Química, Universidad de Caldas, Calle 65 No. 26–10, Manizales 170004, Caldas, Colombia; yaned.correa@ucaldas.edu.co; 2 Departamento de Química, Universidad de los Andes, Carrera 1 No. 18 A–12, Bogotá D.C. 111711, Colombia; 3 Departamento de Química, Universidad Nacional de Colombia, Sede Bogotá. Carrera 30 No. 45–03, Bogotá D.C. 11001, Colombia; lgiraldogu@unal.edu.co

**Keywords:** adsorption, emerging pollutant, carbonaceous material, agroindustrial residues

## Abstract

Caffeine and diclofenac are molecules with high human intake, and both belong to the ‘emergent’ class of contaminants. These compounds have been found at different concentrations in many sources of water worldwide and have several negative impacts on aquatic life systems; that is why the search for new alternatives for their removal from aqueous media is of transcendental importance. In this sense, adsorption processes are an option to attack this problem and for this reason, biochar could be a good alternative. In this regard, were prepared six different biochar from fique bagasse (FB), a useless agroindustry by-product from fique processing. The six biochar preparations were characterized through several physicochemical procedures, while for the adsorption processes, pH, adsorption time and concentration of caffeine and diclofenac were evaluated. Results showed that the biochar obtained by pyrolysis at 850 °C and residence time of 3 h, labeled as FB850-3, was the material with the highest adsorbent capacity with values of 40.2 mg g^−1^ and 5.40 mg g^−1^ for caffeine and diclofenac, respectively. It was also shown that the experimental data from FB850-3 fitted very well the Redlich–Peterson isotherm model and followed a pseudo-first and pseudo-second-order kinetic for caffeine and diclofenac, respectively.

## 1. Introduction

It is normal to evaluate water quality in relation to nutrients, pollutants such as metals, pesticides, hydrocarbons and microorganisms. Nevertheless, advancement and improvement in analytical chemistry techniques has allowed for the detection, identification and quantification of a new group of pollutants, named emerging contaminants. These molecules have been found at low concentrations and come from domestic waste, agroindustry residues and medical waste, among others. Emerging contaminants have various chemical structures and they can be of natural or synthetic origin [1,2]; caffeine (CFN) and diclofenac (DCF) are classified into this group [3].

Caffeine is an alkaloid employed as a stimulant and it can also be used as an additive in drugs to improve their medicinal effect. In addition, it is a resilient molecule and its occurrence in water often occurs as a result of the disposal of home waste [4]. Caffeine has an anxiogenic effect in both wild-type and leopard zebrafish populations [5]. On the other hand, diclofenac is a non-steroidal anti-inflammatory drug (NSAID) that is widely used to treat inflammation and pain [6]. This molecule can cause tissue damage in various mussel species and cytological alterations in rainbow trout. Low concentrations of DCF can lead to undesirable effects on human life, flora, and fauna [7,8]. Different amounts of caffeine and diclofenac in diverse aquifers in many countries around the world have been reported, such as: (i) surface waters: not detected (ND)—1.12 μg L^−1^ and ND—1.04 μg L^−1^ for caffeine and diclofenac, respectively; (ii) groundwater: 0.01–0.17 μg L^−1^ for caffeine and 0–3.05 μg L^−1^ for diclofenac. In addition, they were also found in (iii) conventional wastewater treatment plants (WWTPs) at concentrations of: 0.220–209 μg L^−1^ for caffeine in influent and ND—43.5 μg L^−1^ in effluents; and for diclofenac ˂ 0.001–94.2 μg L^−1^ in influent and ˂ 0.001–0.690 μg L^−1^ in effluents [9]. Furthermore, caffeine and diclofenac were detected at concentrations of 5.50 μg L^−1^ and 21.6 μg L^−1^, respectively, from a wastewater treatment plant from a rural region and before the intake of the supply system in Pereira (Colombia) [10].

To remove contaminants from water, different techniques are used, including filtration, disinfection, coagulation, reverse osmosis, photochemical processes, and advanced oxidation processes. However, in many cases, these processes do not remove emerging contaminants, and therefore it is necessary to apply alternative processes. In this context, adsorption employing carbonaceous material has been evaluated by numerous researchers. For example, carbon fibers, carbon xerogel, carbon nanotubes, graphite, active carbon and other low-cost materials such as biochar have been used to remove caffeine and diclofenac from water. These previous works have shown a wide range of adsorption capacity for caffeine (50.90–1000 mg L^−1^) and diclofenac (0.5260–500.0 mg L^−1^) [8,11,12,13,14,15,16,17,18,19].

Biochar is a carbonaceous material obtained by the pyrolysis of biomasses such as wood waste, sewage sludge, plant leaves, and crop residues, using an inert atmosphere (oxygen-free). Biochar have been employed to improve soil quality, reduce gas emissions and for carbon sequestration. In the first use listed above, it has been employed for more than 2000 years [20,21,22]. For instance, in Terra Preta de Indio (a region in the Brazilian Amazon), the territory has dark earth which is highly fertile for agriculture, and it has been hypothesized that this is due to biochar employment by native residents over a long period of time [21]. In relation to the second use mentioned, reducing gas emissions is possible because biochar is a stable material, so when biochar is mixed with soil, carbon is stored for several hundreds of years, becoming recalcitrant carbon with great resistance to decomposition, and thus decreasing greenhouse gas emissions [23]. Finally, it has been proven that biochar can remove inorganic and organic pollutants such as heavy metals, pigments, pharmaceuticals, aromatic and polyaromatic hydrocarbons, and pathogenic organism from aqueous solutions [24,25]. In this sense, the maximum adsorption capacity of caffeine according to the Langmuir model with a MgAl-layered double hydroxide/biochar composite and oxidized biochar from pine needles was 26.22 mg g^−1^ and 5.350 mg g^−1^, respectively. Furthermore, with modified biochar from *Moringa oleifera* seeds and a composite with MgAl-layered double hydroxide supported on *Syagrus coronata* biochar, the maximum adsorption capacity of diclofenac by the Langmuir model was 121.1 mg g^−1^ and 116.5 mg g^−1^, respectively [26,27,28,29]. Applications of biochar have been increasing because of its features, including: surface area, pore volume, pore size, pH, cation exchange capacity (CEC), electrical conductivity (EC) and surface functional groups, as well as its sustainability, easy production process, and low cost [30,31,32].

Fique bagasse is a useless agricultural residue obtained after extraction of fique fibers, which are the only material of the plant used for manufacturing different products. Therefore, other components (including juices and bagasse) are an environmental problem for farmers that work with fique. It is reported that 93,400 ton of fique bagasse are produced per year. Fique bagasse is 17% (*w*/*w*) of the total of fique leaves and they are mainly composed of cellulose, hemicellulose and lignin at percentages of 42% (*w*/*w*), 22% (*w/w*) and 16% (*w/w*), respectively. The amounts of carbon, hydrogen, oxygen and nitrogen are 36% (*w/w*), 6.0% (*w/w*), 48% (*w/w*) and 1.0% (*w/w*) [33,34,35]. A bibliographic review about the uses of fique bagasse allowed us to determine that this material has not been yet employed to obtain carbonaceous materials. Considering the amount of carbon in fique bagasse and the need to find novel uses for this raw material to reduce the contamination of land and water sources, we assume that obtaining biochar from fique bagasse is a possibility to eliminate or to reduce this agricultural waste.

The objective of this research is to evaluate the adsorption capacity of six different biochar obtained from fique bagasse for remotion of the emerging contaminants caffeine and diclofenac, two pharmaceuticals widely used and frequently detected in wastewater and surface water. Biochar were prepared and characterized by proximate and ultimate analysis, scanning electron microscopy with energy dispersive X-ray spectroscopy (SEM-EDS) and Brunauer, Emmett and Teller (BET) analysis. The adsorption processes and adsorbent–adsorbate interactions were evaluated using equilibrium adsorption data by various kinetic and isotherm models.

## 2. Results and Discussion

### 2.1. Biochar Characterization 

Results of elemental and proximate analysis of fique bagasse biochar (FBB) prepared are shown in Table 1. As shown in Table 1, an increase in the pyrolysis temperature led to a decrease in the percentage of fixed carbon content, whereas the percentage of ash content raised with increasing carbonization temperature of the FBB evaluated. There is evidence that the fixed carbon amount of a biochar depends on feedstock and increases in pyrolysis temperature; nevertheless, this trend changed with increasing ash content in biochar. This was evidenced by biochar obtained at temperatures between 300–600 °C from poultry manure and waste food, in which the amount of fixed carbon ranged from 6.50–0% (*w/w*) and 31.3–13.6% (*w/w*), and the quantity of ash was 46.7–55.8% (*w/w*) and 23.3–52.0% (*w/w*), respectively [36]. Therefore, a decrease in fixed carbon and the percentage of carbon of fique bagasse are shown in Table 1. These data are in coherence with the high percentage of ash in the fique bagasse obtained. In addition, a raise in ash content was directly related to an increase in mineral content and destructive volatilization of lignocellulose matter [37]. This behavior can be related to pH and the Point of Zero Charge (PZC) of the FBB analyzed, since increasing the basicity can be attributed to the presence of metals (including potassium, calcium and magnesium) and a decrease in functional groups such as hydroxyl (-OH) and carboxyl (-COOH) in their structure, which was confirmed by infrared spectroscopy studies [38].

**Figure 1:**
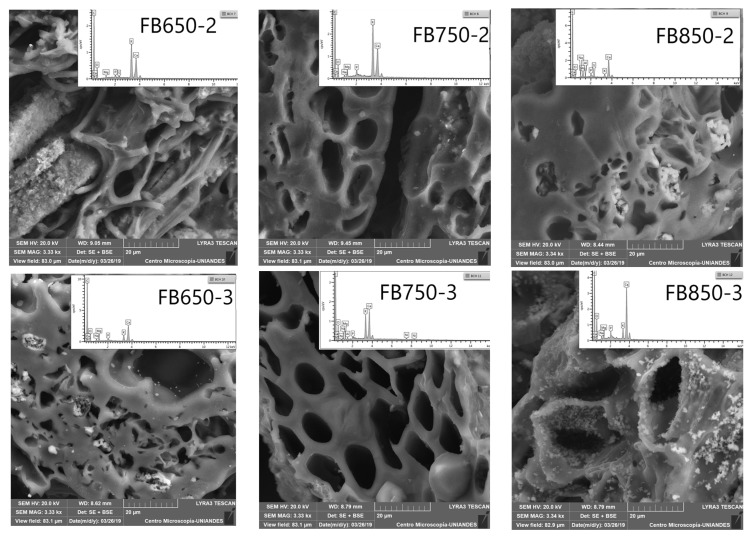
Scanning electron microscopy (SEM) images of fique bagasse biochar surface, including the energy dispersive X-ray (EDX) spectra.

Furthermore, SEM-EDS analysis (Figure 1) showed the presence of alkaline metals (K, Ca, Mg); it should be noted that the amount of these elements was not equal on the surface of the FBB analyzed, and it increased with pyrolysis temperature. Such results were also obtained for previously analyzed FBB [39]. Finally, calcium and magnesium were quantified in fique fibers, tow and pulp [40]. They were still present in FBB as they did not disappear during the pyrolysis process. On the other hand, SEM showed a heterogeneous surface and honeycomb-like porous structures in all prepared FBB. These shapes originated from heterogeneous veins, lateral pits, and helical fibrils from the original native tissue structure in bagasse [41] (Figure 1). Additionally, by elemental analyses, high carbon and oxygen levels with small hydrogen and nitrogen quantities were determined; furthermore, sulfur was not detected in FBB (Table 1). This result was similar to those obtained for other biochar from different sources. Moreover, the results did not show any significant changes for carbon, oxygen and hydrogen contents, except for FB850-3. This biochar had a lower amount of carbon and higher oxygen content than the other biochar evaluated. This result is contrary to what was presented by other researchers [41,42,43] and this is possible because of the precursor structures and the mineral constituents of fique bagasse.

**Figure 2:**
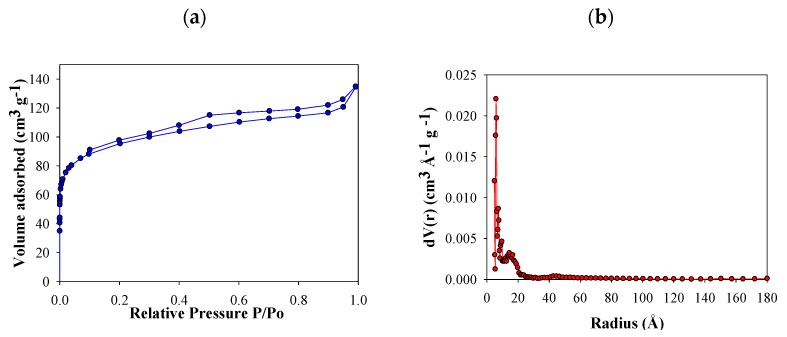
N_2_ adsorption–desorption isotherm of FB850-3 (**a**) and pore size distributions by Quenched Solid Density Functional Theory (QSDFT) of FB850-3 (**b**).

N_2_ adsorption isotherms of FB850-3 are presented in Figure 2a. Based on International Union of Pure and Applied Chemistry (IUPAC) classification, this was categorized as a type IV curve—this curve type is characteristic of micro mesoporous solids [44]. At a relative pressure of around 0.10, FB850-3 pores were filled, which is associated with the monolayer formation of N_2_ adsorbed into the micropores. Then, there is the formation of multilayers and filling of the mesoporous. The hysteresis is associated with capillary condensation of nitrogen inside the pores. In addition, a variable microporosity in the size range of 1–20 Å is observed, which shows a narrow pore size distribution (Figure 2b).

The behavior of FB850-3 does not follow the trend of the other five fique bagasse biochar obtained in this work, in which a minimum increase in area with increasing temperature and residence time was obtained (Table 1). It is known that pyrolysis of lignocellulosic materials involves a series of reactions; therefore, the experimental changes under different heating conditions affect this process [45]. In the case of fique bagasse, thermogravimetric analysis showed slow decomposition between 600–900 °C. In this interval of temperature, volatile molecules are released according to the literature. In this sense, it could be assumed that with residence times of 1 and 2 h, char formation can be inhibited or autocatalysis could occur, and laevoglucosan is formed de novo [46]. When the residence time is increased to 3 h, the volatiles are allowed to escape from the char, and this increases the porosity and surface area of FB850-3.

### 2.2. Effect of pH on Adsorption

**Figure 3:**
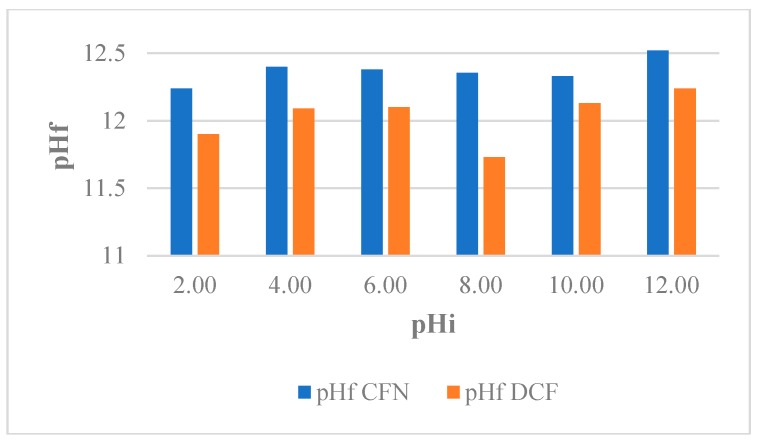
Variation pH of caffeine (CFN) and diclofenac (DCF) solutions evaluated for FB850-3.

pH-induced changes in the physicochemical properties as well as the adsorbate behavior have been reported. In addition, depending on the pH, the adsorbate is superficially charged and this can affect the interaction with the adsorbent [47]. Therefore, the effect of pH on the capacity of adsorption of FBB for the removal of CFN and DCF was evaluated and the results showed insignificant differences for the pH intervals analyzed [38]. This effect may be due to the basic minerals present in the resulting slurries when CFN and DCF solutions were mixed with the biochar samples, which made the pH of the slurries at the end of process similar (pH*_f_* = 12.00 ± 0,4), regardless of the initial pH (Figure 3). For this reason, all experiments were performed in solutions of pH 5.90 ± 0.18 (CFN) and pH 6.80 ± 0.24 (DCF). Under these conditions, the structure of CFN was neutral, the structure of DCF was ionic, and surface of the biochar were neutral.

### 2.3. Adsorption Kinetics

**Figure 4:**
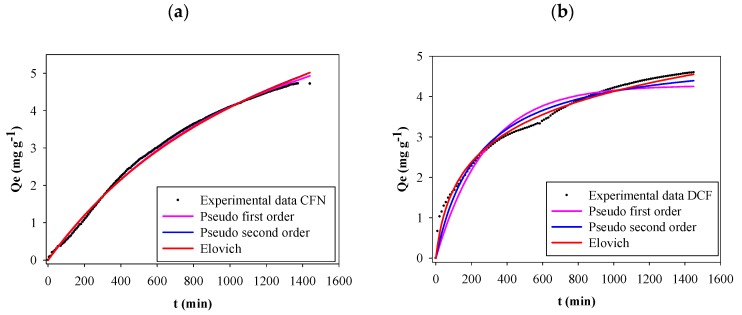
Kinetics adsorption of CFN onto FB850-3 by fitting the pseudo-first-order, pseudo-second-order and Elovich model (**a**), adsorption kinetics of DCF onto FB850-3 by fitting the pseudo-first-order, pseudo-second-order and Elovich model (**b**).

The effect of contact time on FB850-3 adsorption capacity of CFN and DCF was evaluated over 24 h. Figure 4 shows that CFN or DCF adsorption increased rapidly at the initial contact time and then adsorption decreased after longer contact times. Finally, equilibrium was obtained at 24 h where the adsorption capacities were 4.72 mg g^−1^ and 4.60 mg g^−1^, for CFN and DCF, respectively. A concentration gradient on the active surface of the biochar might cause the rapid adsorption at the beginning of the process [47,48]. From the above result, a 24 h period was selected for all experiments.

Table 2 shows all the kinetic model parameters, correlation coefficients and several errors determined with experimental data for CFN and DCF adsorption onto FB850-3. Figure 4 presents kinetic plots for CFN and DCF adsorption on FB850-3. It was evident that the three graphs showed the same trend; however, the correlation coefficient from the CFN and DCF kinetic adsorption model evaluated showed that the pseudo-first-order kinetic model was the best one of the three analyzed for CFN. This model proposed that the rate of interaction of adsorption depends on the number of free sites in the adsorbent [49]. In addition, the pseudo-second-order kinetic model was the best one for DCF, and the Elovich model showed the lowest values of statistical indices of the three models evaluated. This allows us to suggest that there is a chemisorption process of DCF onto FB850-3; moreover, the surface of FB850-3 is heterogeneous [49,50]. Finally, the adsorption capacity calculated from the Elovich model was similar with the experimental adsorption capacity of the biochar at equilibrium (Qe) value.

Contrary to the results of this research, the kinetic adsorption of caffeine onto biochar obtained from *Eichhornia crassipes* showed that the experimental data fitted well with the pseudo-second-order model [51]. Also, this model was the best for the kinetic adsorption of CFN onto activated carbon fibers (ACFs) from pineapple plant leaves [11], and in the kinetic adsorption of CFN onto char from the gasification of coal and pine activated with K_2_CO_3_ [52]. Furthermore, the experimental data of the kinetic adsorption of CFN onto activated carbons from coco and babassu coco and a commercially available activate carbon (NO: Norit1 Granular Activated Carbon (GAC) 1240 plus) were appropriately described by a pseudo-second-order kinetic model [53]. In addition, adsorption of CFN and diclofenac by carbon xerogels fitted well for a pseudo-second-order model [54]. Moreover, in relation to DCF, the pseudo-second-order kinetic model was a better fit for active carbon from tea waste [15], and purified multi-walled carbon nanotubes [16]. Finally, biochar obtained from pinewood fitted well with the pseudo-first-order kinetic model and biochar from pig manure followed the pseudo-second-order kinetic model [12].

### 2.4. Adsorption Isotherm

**Figure 5:**
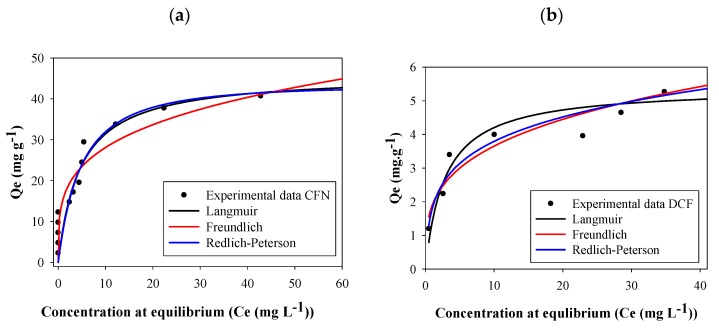
Adsorption isotherms of caffeine (**a**) (CFN) and diclofenac (**b**) (DCF) onto FB850-3.

The adsorption capacity was high at low concentrations of both caffeine and diclofenac. Nevertheless, the plateau of the graph was obtained at a lower concentration for diclofenac than for caffeine (Figure 5). According to the Giles classification, these isotherms are L2-type; this type of isotherm indicates a high affinity between the adsorbent and the adsorbate [55].

Parameters of all isotherm models calculated with the experimental adsorption data for CFN and DCF are reported in Table 3. In this table, it can be seen that higher correlation coefficient (R^2^) values were obtained using the Redlich–Peterson model. This isotherm assumes a hybrid adsorption mechanism, since it is a combination between the Langmuir and Freundlich models. If exponent B of the Redlich–Peterson equation is close to 1, the Langmuir will be the suitable isotherm, whereas if B is near to 0, to facilitate Henry’s law and insights, the Freundlich model will be a better option [17,56]. In this work, caffeine B values vary between 1.07–0.165; the last value was obtained for FB850-3 which suggests that Freundlich will be the best isotherm for this biochar. In addition, diclofenac B values ranged between 1.23–0.506, indicating that for all fique bagasse biochar evaluated, the adsorption behaviors will be related with the Langmuir equation. The separation factors (R_L_) for CFN and DCF were thus calculated from the Langmuir isotherm. The R_L_ values were ˂ 1 for all biochar analyzed in this work, suggesting that all adsorption processes by FBB were favorable [49]; furthermore, FB850-3 was the most favorable one.

It has been reported that carbonaceous materials can adsorb molecules due their physical and chemical properties. In this sense, the results of the fique bagasse biochar evaluated in this study showed that a direct correlation exists between the available surface area of fique bagasse biochar and the maximum caffeine and diclofenac adsorption capacity; therefore, it is possible to assume that this physical property permitted the retention of CFN and DCF in this carbonaceous material. In addition, the porous structure of FB850-3 favored more diffusion of caffeine than diclofenac due the smaller molecular dimensions of CFN (0.98 × 0.87 nm) compared to DCF (0.97 × 0.96 nm) [3]. Furthermore, several researchers have reported that carbonaceous materials can adsorb aromatic molecules through π–π stacking or hydrophobic interactions [43,57]. For example, Tan et al. explained that the adsorption of caffeine and diclofenac onto a carbon surface take place by parallel relations between aromatic rings of these organic molecules and the adsorbent [58]. This previous information allows us to suggest that caffeine and diclofenac adsorption onto the biochar evaluated was caused by π−π electron-donor−acceptor complex interactions between the aromatic ring structures of CFN and DCF and the studied fique bagasse biochar.

Comparing the Qmax parameter of the Langmuir model for caffeine and diclofenac adsorption obtained in this study (40.20 mg g^−1^ and 5.40 mg g^−1^ for FB850-3, respectively) with other research, it is evident that values obtained in this work were higher than that reported by Correa et al., Ortiz-Martínez et al., and Lonappan et al. [12,39,59]. In contrast, they were lower compared to other adsorbents and conditions tested (Table 4). This shows that for improving the adsorption of pollutants by carbon-based materials, it is necessary to perform chemical or physical modification of the adsorbents.

**Figure 6:**
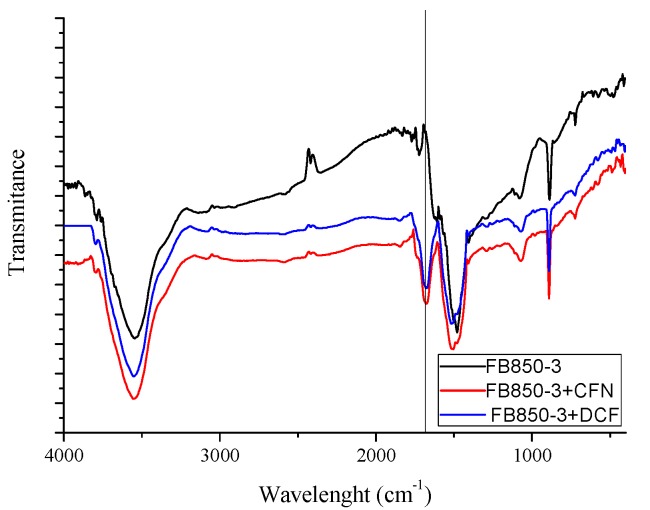
Infrared spectrum of FB850-3 prior to and post adsorption of CFN or DCF.

IR spectrums of FB850-3+CFN and FB850-3+DCF (Figure 6) show one band at 1700 cm^−1^; this band is related to the stretching of C=N or C=O [65]. These functional groups are associated with CFN or DCF molecules, confirming that CFN and DCF were adsorbed onto the surface of FB850-3. 

**Figure 7:**
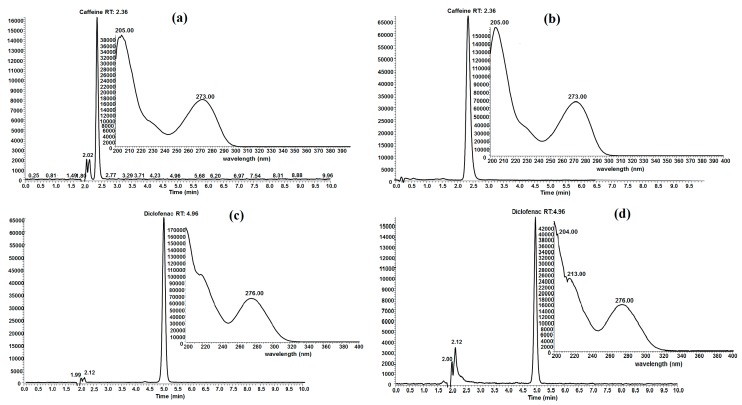
HPLC chromatogram and UV spectrum of caffeine (**a**), FB850-3+CFN (**b**), diclofenac (**c**) FB850-3+DCF (**d**).

Experimental conditions and the biochar employed for adsorption capacity determination could cause chemical structure modification of caffeine and diclofenac and it is possible that some other molecules of decomposition are produced which do not absorb in the same wavelength that caffeine and diclofenac absorb. Therefore, evaluation by UV-VIS spectrophotometry at the end of the adsorption process could give false data. To demonstrate that neither caffeine nor diclofenac are being modified in this work, high performance liquid chromatography (HPLC) analyses were performed. The evaluation of aqueous solutions of FB850-3+CFN and FB850-3+DCF by HPLC showed that both emerging contaminants, CFN and DCF, did not undergo structure modification during the adsorption process (Figure 7). This was evident as the retention time of the elution and ultraviolet spectrum were the same in both standards of CFN and DCF and aqueous samples of FB850-3+CFN and FB850-3+DCF.

## 3. Materials and Methods

### 3.1. Reagents

All reagents used were analytical grade. Caffeine (CFN) and diclofenac sodium (DCF) were purchased from Merck (Darmstadt, Germany) and Sigma-Aldrich (Steinheim, Germany), respectively. CFN and DCF stock solutions at 500 mg L^−1^ were used to prepare the different dilutions employed in all experiments.

### 3.2. Biochar Preparation

Fique bagasse (FB) was collected in a farm in Aranzazu (Caldas, Colombia) after fiber extraction. Fiber extraction is a craft process in which a hand machine is used throughout where leaves of fique are squeezed to obtain fiber, juices and bagasse. Fique bagasse (FB) was dried at 100 °C for 48 h in a furnace oven (Thelco Laboratory Oven, Vernon Hills, IL, USA). Following this, it was sieved through a 100 mm sieve and finally, six types of biochar were produced by combining three temperatures (650 °C, 750 °C, and 850 °C) and two residence times (120 min and 180 min) using a horizontal furnace (Thermo Scientific Thermolyne, Wertheim, Germany) in the presence of nitrogen at 100 mL min^−1^ (to generate an oxygen free atmosphere). For each run, the heating rate was fixed at 1 °C min^−1^. These samples were coded as FB650-2, FB750-2, FB850-2, FB650-3, FB750-3 and FB850-3, respectively.

### 3.3. Biochar Characterization

Ultimate analysis (CHNS) of the biochar produced were evaluated with an organic elemental analyzer (Thermo Scientific Flash 2000, Milano, Italy). The oxygen contents were calculated by difference. Proximate analysis (including fixed carbon, volatile matter and ash contents) were determined by the American Standard Test Method D 7582-15 [66].

Surface morphology and chemical composition of different biochar prepared were obtained by scanning electron microscopy (SEM) using a JSM-6490LV with an X-ray scattered energy detector (SED) (JEOL, Peabody, USA). Biochar samples were gold coated prior to viewing by SEM [41]. Surface area (S_BET_) was obtained by the Brunauer, Emmet and Teller (BET) equation, employing N_2_ adsorption using an Autosorb iQ instrument (Quantachrome instruments, Boynton Beach, USA). Micropore volume and pore size distribution (PSD) were determined by Dubinin–Radushkevich and by the Barrett–Joyner–Halenda (BJH) models, respectively [67]. pH was measured by preparing a suspension of 10 mg of biochar in 20 mL of deionized water, which was stirred for 24 h prior to the pH being measured with a pH meter (Hanna Instruments, HI 2211, Leighton Buzzard, UK) [67]. The superficial functional groups on the produced biochar were analyzed by FTIR (Shimadzu, IR Tracer-100, Kyoto, Japan). Samples were mixed with KBr and kept in an oven at 105 °C for 24 h [68].

### 3.4. Adsorption Experiments

For all experiments, 50 mg of biochar was placed in a flask containing 5 mL solution of known CFN or DCF concentration. All experiments were performed at solutions of pH 5.90 ± 0.18 (CFN) and 6.80 ± 0.24 (DCF) and shaken at 200 rpm at 20 °C. After that, the concentrations of CFN or DCF were obtained carefully by a calibration curve using UV-Vis spectrophotometer (Thermo Spectronic Genesys 5, Waltham, USA). The effect of contact time (0–24 h), initial concentration CFN (25–200 mg L^−1^) or DCF (12.5–100 mg L^−1^) were evaluated. The adsorption capacity of the adsorbent at any time (Qt, mg g^−1^) and at equilibrium (Qe, mg g^−1^), were obtained from Equation (1). All studies were carried out in triplicate.

(1) $$Q_{e}=\frac{V\left(Co-Ce\right)\;\;}{W}$$

where *Co* is the initial concentration of CFN or DCF (mg L^−1^), *Ce* is the concentration of CFN or DCF at equilibrium, *V* (L) is the volume of CFN or DCF solution and *W* (g) is the dry mass of the biochar used [69].

### 3.5. Effect of pH 

Caffeine and diclofenac adsorption capacity onto biochar at different pH were performed at five different pH: 2.0, 4.0, 6.0, 8.0 and 10.0 for CFN, and three different pH for DCF: 6.0, 8.0 and 10.0. For this assay, 50 mg of biochar with 5 mL of 50 mg L^−1^ of CFN and 20 mg L^−1^ of DCF were used. The slurries were shaken at 200 rpm for 24 h at 20 °C. Finally, for each assay, the pH of the slurries were measured.

### 3.6. Adsorption Kinetics

After evaluating the effect of pH on the adsorption capacity of all biochar obtained, it was determined that the only biochar with significant remotion of CFN and DCF was FB850-3. Then, we performed kinetic assays with this carbonaceous material. 

Kinetic assays were performed by adding 0.03 g of FB850-3 into 0.003 L of caffeine or diclofenac solution at 50 mg L^−1^ and 75 mg L^−1^, respectively. The solution was placed in a quartz cell for 24 h and spectrometer software of the UV-Vis spectrophotometer (Thermo Spectronic Genesys 5, Waltham, USA) allowed us to obtain data for kinetic analysis at regular intervals. Then, by using Equation (1), we determined the adsorption capacity of FB850-3 for CFN and DCF.

Three kinetic models were analyzed to determine which one of the mechanisms of adsorption was prevalent: pseudo-first-order (Equation (2)), pseudo-second-order (Equation (3)) and Elovich (Equation (4)). The equations were used with experimental data and normalized standard deviation (Δq(%)), chi-square (χ^2^), average relative error (ARE(%)) and hybrid fractional error function (HYBRID), were calculated using Equations (5–8); these tests were employed to determine the errors in the experimental data, and their values should be as close to ‘zero’ as possible [47].

(2) $$Q_{t}=Q_{e}(1-\exp\left(-kt\right)$$

(3) $$Q_{t}=\frac{Q_{e}^{2}k_{2}t}{1+Q_{e}k_{2}t}$$

(4) $$Q_{t}=\frac{1}{\beta }\ln\left(1+\alpha \beta t\right)$$

where *Q_t_* (mg g^−1^) is the adsorption capacity at time *t*; *t* is the time (min), *Q_e_* (mg g^−1^) is the adsorption capacity at equilibrium, *k* is the rate constant of the pseudo-first-order model (1 min^−1^), *k*_2_ (g mg^−1^ min^−1^) is the pseudo-second-order rate constant, α (mg g^−1^ min^−1^) and β (g mg^−1^) are the constants for the Elovich model.

(5) $$\Delta q\left(\%\right)=100\sqrt{\frac{1}{N-1}\overset{N}{\underset{i=1}{\sum }}{\left(\frac{\left(Qexp-Qcal\right)}{Qexp}\right)}_{i}^{2}}$$

(6) $$ARE\left(\%\right)=\frac{100}{N-1}\overset{N}{\underset{i=1}{\sum }}{\left(\frac{\left(Qexp-Qcal\right)}{Qexp}\right)}_{i}^{2}$$

(7) $$\chi^{2}=\overset{N}{\underset{i=1}{\sum }}\left(\frac{{\left(Qexp-Qcal\right)}^{2}}{Qcal}\right)$$

(8) $$HYBRID=\frac{100}{N-p}\overset{N}{\underset{i=1}{\sum }}{\left(\frac{\left(Qcal-Qexp\right)}{Qexp}\right)}_{i}^{2}$$

where *Qexp* and *Qcal* are the experimental value and the calculated value for adsorption capacity of biochars evaluated at time ‘t’ or equilibrium concentration, respectively, *N* is the number of measurements, and *p* is the number of parameters in each model.

### 3.7. Adsorption Isotherm

In order to comprehend the probable adsorption mechanism implicated in this process, three models have been evaluated in this study: the Langmuir (Equation (9)), Freundlich (Equation (10)), and Redlich–Peterson (Equation (11)) models. The models were used to analyze experimental equilibrium data and the adjustment quality was evaluated through the determination coefficient (R^2^) [56,70,71].

(9) $$Qe=\frac{QoK_{L}Ce}{1+K_{L}Ce}$$

(10) $$Qe=K_{F}Ce^{1/n}$$

(11) $$Qe=\frac{K_{RP}Ce}{1+a_{R}Ce^{B}}$$

where *Qe* (mg g^−1^) is the equilibrium CFN or DCF concentration in the biochar, *Ce* (mg L^−1^) is the equilibrium concentration of CFN or DCF in the aqueous phase, *Qo* (mg g^−1^) is the maximum adsorption, and *K_L_* (L g^−1^) is the Langmuir equilibrium adsorption constant. *K_F_* (L g^−1^) is the Freundlich constant or relative sorption capacity, and *n* is a constant indicating adsorption intensity. *K_RP_* (L g^−1^) and *a_R_* (L mg^−1^) are Redlich–Peterson isotherm constants, and *B* is the exponent.

### 3.8. Chromatographic Analysis 

For caffeine and diclofenac evaluation after adsorption onto fique bagasse biochar, liquid chromatographic analysis were carried out on a UltiMate 3000 UHPLC (Thermo Fisher Scientific, Waltham, MA, USA). A Prontosil C18 column (4.0 mm × 125 mm, 5 μm, 100 Å) (Bischoff Chromatography, Leonberg, Germany) was used at 40 °C. The mobile phase was A (0.1% aqueous formic acid) and B (acetonitrile (0.1% formic acid) at 4:6 and a flow of 0.5 mL min^−1^. Detection was performed at 274 nm and 273 nm, and Diode Array Detector (DAD) was between 190–290 nm. 

## 4. Conclusions

Biochar obtained from fique bagasse is an alternative carbonaceous material to capture contaminants from water. Moreover, to obtain this biochar, we used a lignocellulosic residue which is an environmental problem in the production of fique fiber in Colombia. Therefore, biochar could be an option to employ this agroindustrial waste. The caffeine (CFN) and diclofenac (DCF) adsorption capacity was gradually increased when temperature and residence time for biochar production was increased (650 °C, 750 °C and 850 °C; 2 h and 3 h), with FB850-3 being the biochar with the highest adsorption capacities for CFN and DCF (40.20 mg g^−1^ and 5.40 mg g^−1^, respectively). This behavior was in accordance with their surface area (212 m^2^ g^−1^). These results suggest that the porous structure of FB850-3 allowed more appropriate pore-filling for CFN than for DCF adsorption. In addition, molar ratios of H/C show that increasing pyrolysis temperatures induced the formation of aromatic sheets/clusters in fique bagasse biochar and these aromatic structures can contribute to π–π electron donor–acceptor interactions between biochar and CFN or DCF. Finally, alkaline metals (K, Ca and Mg) present in fique bagasse biochar always resulted in the final pH of the evaluated slurry being basic, and this result did not show any effect of the pH on the removal of CFN and DFC by fique bagasse biochar in these experiments.

## Figures and Tables

**Table 1:** Brunauer, Emmett and Teller surface area (SBET) and elemental analysis of the obtained fique bagasse (FB) biochar.

Sample	S_BET_ (m^2^ g^−1^)	pH ^a^	Elemental Analysis (%)					Proximate Analysis (%)		
			C	O ^b^	S	N	H	Fixed Carbon	Volatile Matter	Ash Content
FB650-2	0.8420	11.40	52.207	44.973	nd	1.7870	1.0328	66.82	4.120	29.06
FB750-2	1.937	11.24	51.061	46.262	nd	1.7631	0.9136	66.14	3.020	30.84
FB850-2	5.351	11.38	51.242	46.390	nd	1.4967	0.8718	67.88	2.160	29.96
FB650-3	2.162	11.14	54.127	42.810	nd	1.8955	1.1672	72.40	0.9800	26.62
FB750-3	8.432	11.74	53.539	43.573	nd	1.9080	0.9794	68.96	2.190	28.85
FB850-3	211.7	11.35	43.644	54.340	nd	1.1457	0.8696	61.42	2.900	35.68

^a^ pH slurry, ^b^ by difference. nd: not detected.

**Table 2:** Parameters of different kinetic models and statistical indices for the adsorption of caffeine and diclofenac onto FB850-3 at 20 °C.

Model	Parameter	Caffeine	Diclofenac
	*Q_e exp_* (mg g^−1^)	4.73	4.07
Pseudo-first-order	*Q_e cal_* (mg g^−1^)	4.94	4.25
	*k* (1 min^−1^)	0.000800	0.00300
	R^2^	0.990	0.980
	Δq (%)	7.11	14.5
	ARE (%)	3.09	8.26
	χ^2^	0.750	7.69
	HYBRID	0.220	2.40
Pseudo-second-order	*Q_e cal_* (mg g^−1^)	5.02	4.39
	*k*_2_ (1 min^−1^)	0.0000600	0.000800
	R^2^	0.910	0.990
	Δq (%)	7.36	11.1
	ARE (%)	4.21	5.40
	χ^2^	0.890	3.67
	HYBRID	0.410	1.80
Elovich	*Q_e cal_* (mg g^−1^)	5.10	4.55
	*α* (mg g^−1^ min^−1^)	0.00700	0.0390
	β (g mg^−1^)	0.240	0.860
	R^2^	0.810	0.950
	Δq (%)	7.57	6.82
	ARE (%)	4.76	2.70
	χ^2^	0.00800	0.100
	HYBRID	0.530	0.640

*Q_e exp_* and *Q_e cal_* are the experimental value and the calculated value for adsorption capacity of biochar evaluated at equilibrium time, respectively, *k* is the rate constant of the pseudo-first-order model (1 min^−1^), *k*_2_ (g mg^−1^ min^−1^) is the pseudo-second-order rate constant, α (mg g^−1^ min^−1^) and β (g mg^−1^) are the constants for the Elovich model. (R^2^) is the determination coefficient, (Δq(%)) is normalized standard deviation, ARE (%) is average relative error, (χ^2^) is chi-square and (HYBRID) is hybrid fractional error function.

**Table 3:** Parameters of the Langmuir, Freundlich and Redlich–Peterson isotherm models for caffeine and diclofenac adsorption onto fique bagasse biochar at 20 °C.

Sample		Freundlich			Langmuir			Redlich–Peterson			
		Kf (L g^−1^)	n	R^2^	Qmax (mg g^−1^)	K (L mg^−1^)	R^2^	K_RP_ (L g^−1^)	a_R_ (L mg^−1^)	B	R^2^
**FB650-2**	**CFN**	0.152	0.19	0.86	0.4465	0.0607	0.95	0.0218	0.0340	1.07	0.96
**FB750-2**		0.0436	0.45	0.97	0.6143	0.0140	0.96	0.0684	1.27	0.584	0.97
**FB850-2**		0.108	0.05	0.83	1.711	0.288	0.93	24.6	49.7	0.712	0.93
**FB650-3**		0.0182	0.60	0.95	0.7619	0.00630	0.93	0.374	20.1	0.402	0.95
**FB750-3**		0.0902	0.46	0.89	1.317	0.0145	0.95	0.0487	0.255	0.660	0.91
**FB850-3**		15.3	0.26	0.82	40.20	0.216	0.85	9.02	1.04	0.165	0.85
**FB650-2**	**DCF**	0.0442	0.28	0.98	0.1802	0.0124	0.92	2.79	62.9	0.716	0.98
**FB750-2**		0.0664	0.19	0.97	0.1704	0.0209	0.98	0.0317	0.270	0.921	0.99
**FB850-2**		0.0292	0.52	0.93	0.4643	0.00910	0.93	0.100	2.97	0.506	0.93
**FB650-3**		0.0755	0.25	0.84	0.2583	0.0211	0.96	0.0139	0.0179	1.23	0.99
**FB750-3**		0.0868	0.27	0.94	0.3185	0.0810	0.89	0.909	10.2	0.738	0.94
**FB850-3**		1.90	0.28	0.91	5.402	1.89	0.88	6.93	2.85	0.781	0.92

*Qmax* (mg g^−1^) is the maximum adsorption and *K_L_* (L g^−1^) is the Langmuir equilibrium adsorption constant. *K_F_* (L g^−1^) is the Freundlich constant or relative sorption capacity, and *n* is a constant indicating adsorption intensity. *K_RP_* (L g^−1^) and *a_R_* (L mg^−1^) are Redlich–Peterson isotherm constants, and *B* is the exponent.

**Table 4:** Comparison of the adsorption capacity of caffeine and diclofenac considering the Qmax parameter of the Langmuir model with other adsorbents.

Adsorbent	Qmax (mg g^−1^)	pH	Temperature (°C)	Ref
	**Caffeine**			
Carbon fibers from pineapple plant leaves	155.5	5.8	25	[11]
Carbon xerogel (CX) modified with (CH_3_COO)_2_Cu and ethylenediamine	118.0	2.0	25	[19]
CX modified with (CH_3_COO)_2_Cu	107.0			
Peach carbon	250.0	---	30	[6]
Oxidized peach carbon	126.0			
Helium peach carbon	260.0			
F-400 granular activated carbon	190.9	6.3	23	[3]
Grape stalk	89.20	2.0	Room temperature	[13]
Grape stalk modified by phosphoric acid	129.6	2.0		
Activated carbon from grape stalk	916.7	4.0		
Carbon xerogel (CX)	79.10	--	30	[54]
CX in nitric acid	50.90			
CX in urea solution	185.4			
CX in concentrated sulfuric acid	52.60			
Graphite sheet modified by an electrochemical exfoliation/oxidant process	1000	--	--	[60]
Santa Barbara amorphous-15 (SBA-15) mesoporous silica	0.2300	Neutral pH	Ambiental temperature	[59]
SBA-15 modified with Co^2+^	0.07000			
SBA-15 modified with Ni^2+^	0.01000			
SBA-15 modified with Cu^2+^	0.08000			
	**Diclofenac**			
F-400 Granular activated carbon		6.3	23	
Peach carbon	200.0	---	30	[6]
Oxidized peach carbon	198.0			
Helium peach carbon	170.0			
Pine wood biochar	0.5263	6.5	25	[12]
Pig manure biochar	12.50			
Organobentonite	500.5	7.0	25	[8]
Expanded graphite	330.0	--	Room temperature	[61]
Activated carbon from cocoa	63.47	7.0	25	[62]
Regenerable granular carbon nanotubes/alumina hybrid	31.54	6.0	---	[63]
Granular activated carbon	36.23	5.5	25	[17]
Multi-walled carbon nanotubes	19.90	6.0	25	[16]
Graphene oxide	500.0	7.0	20	[18]
Commercial activated carbon Tea waste carbon activated with: K_2_CO_3_ KOH H_2_SO_4_	136.0 91.20 81.96 74.60	6.5	30	[15]
Activated carbon derived from pine tree	54.67	7.0	Room temperature	[64]
Carbon xerogel (CX)	58.50	--	30	[54]
CX in nitric acid	54.00			
CXN in urea solution	140.2			
CX in concentrated sulfuric acid	78.80			
